# Deceased Donor Renal Allograft Utility in Adult Single and Multi-organ Transplantation in the United States

**DOI:** 10.1097/TXD.0000000000001744

**Published:** 2024-12-18

**Authors:** Peter J. Altshuler, Adam S. Bodzin, Kenneth A. Andreoni, Pooja Singh, Anju Yadav, Jaime M. Glorioso, Ashesh P. Shah, Carlo Gerado B. Ramirez, Warren R. Maley, Adam M. Frank

**Affiliations:** 1 Department of Surgery, Division of Transplant Surgery, University of California San Francisco, San Francisco, CA.; 2 Department of Surgery, Division of Transplantation, Thomas Jefferson University Hospital, Philadelphia, PA.; 3 Department of Medicine, Division of Nephrology, Thomas Jefferson University Hospital, Philadelphia, PA.

## Abstract

**Background.:**

Deceased donor multiorgan transplants utilizing kidneys (MOTs) can improve outcomes for multiorgan recipients but reduces kidneys for chronic renal failure patients.

**Methods.:**

We reviewed the Organ Procurement and Transplantation Network database from 2015 through 2019, for adult deceased donor kidney transplants. Recipients were classified as kidney transplant alone (KTA) (n = 62,252) or MOTs pancreas-kidney, simultaneous pancreas-kidney (n = 3,976), liver-kidney, simultaneous liver-kidney (n = 3,212), heart-kidney, simultaneous heart-kidney (n = 808), and “other”-kidney, simultaneous “other” kidney (n = 73).

**Results.:**

Liver, heart, and lung-alone transplants were at least 7 times more frequent than their MOT correlate, whereas the inverse was true with pancreas transplantation with SPKs being by far the most common pancreas transplant type. On average, KTA recipients waited between 2.8 and 21.4 times longer than MOTs, with SPKs waiting the longest of the MOT types. Predialysis initiation transplants were less frequent in KTAs compared with MOTs. Use of high-quality grafts according to Kidney Donor Profile Index < 35% was frequent among MOTs, but uncommon in KTAs who had an Estimated Post Transplant Survival score (EPTS) of >20%. For recipients older than 65, SPKs and SOKs were rare, but SLKs and SHKs had a higher fraction of recipients than KTAs and were much more likely to use a Kidney Donor Profile Index <35% kidney. SPKs and KTAs with an EPTS ≤20% had the best kidney graft survival. KTAs with an EPTS ≤80% had better kidney graft survival than SLKs, SHKs, and SOKs.

**Conclusions.:**

This study highlights disparities in access to deceased donor kidneys for kidney-alone candidates versus MOTs and suggests opportunities to improve allocation.

## INTRODUCTION

Current organ shortages in renal transplantation have resulted in >85,000 adults (≥18 y-old) with advanced or end-stage renal disease awaiting kidney transplant,^1^ with mean waiting time exceeding 4 y and the majority of candidates not accessing transplant.^2^ Multiple policy changes have occurred over the time the Organ Procurement and Transplant Network (OPTN) has been in existence with the goal of maximizing fairness and utility of deceased donor kidney transplant. One of the most important changes that has occurred was the implementation of the Kidney Allocation System (KAS) which took effect December 4, 2014.^3^ KAS improved the state of deceased donor kidney transplantation through 3 main policy alterations. It allowed dialysis time to be captured for prospective candidates who are referred after dialysis initiation, increased access for the most broadly sensitized and blood type B candidates, and initiated longevity matching for the 20% best adult candidates as defined by the Estimated Post Transplant Survival score (EPTS).

KAS relied on using 2 new measurement tools to better characterize deceased donor kidney graft quality and the expected longevity of adult kidney graft recipients. The KDPI score which was derived from the kidney donor risk index was developed incorporating ten donor variables giving a score from 0% to 100% with lower scores representing a superior quality kidney.^3,4^ Similarly, on the recipient side the EPTS was fashioned from the candidate’s age; candidate’s dialysis time; whether the candidate had a prior history of transplant; and whether the candidate is diabetic, and like the KDPI a score from 0% to 100% is given, with lower percentages representing a longer anticipated post-transplant survival.^5,6^

A population in deceased donor kidney allocation that may not be directly impacted by the KAS, but are affected nonetheless, are those candidates that require a simultaneous non-renal organ transplant. Recipients of a non-renal organ with a kidney, or multiorgan transplants utilizing kidneys (MOTs) have largely demonstrated improved overall outcomes in comparison with non-renal recipients who require posttransplant dialysis.^7-9^ The number of MOTs in the United States has been increasing in proportion to the number of overall transplants, with 1,062 MOTs performed in 2000, 1,260 in 2010, 1,471 in 2015, and 1,807 in 2019. Between 2000 and 2019, simultaneous liver-kidney (SLK) and simultaneous heart-kidney (SHK) transplants in particular have increased in frequency, with SLK transplants increasing 439% (135 to 727) and SHK transplants increasing 655% (29 to 219).^2^

While adding a kidney to a non-renal organ transplant may improve outcomes in MOTs, each MOT category is subject to distinct regulations driven by the allocation of the non-renal organ. MOTs decrease the availability of deceased donor kidneys for chronic renal failure alone candidates,^10^ and the OPTN MOT Committee has been instrumental in driving policy creation, implementation, and revision in multiorgan transplantation.^11^ This study set out to further characterize the impact of MOTs on the KTA candidate population, assessing organ-sharing equity, utility, and graft survival in kidney transplantation.

## METHODS

### Patient Population

A retrospective review of adult deceased donor kidney transplants identified in the OPTN database from January 1, 2015, to December 31, 2019, was performed. January 1, 2015, was chosen as the starting date of data accrual owing to the implementation of the United Network for Organ Sharing KAS policy in December 2014, while December 31, 2019, was the last date of accrued data to allow for sufficient graft survival analyses. Specific single and multiorgan transplants identified were KTA, simultaneous pancreas-kidney (SPK), SLK, SHK, and other multi-organ transplants identified as simultaneous “other” kidney (SOK) transplants (intestine, lung, and 3-organ). Multiorgan recipients were identified as MOTs if their kidney transplant occurred within 2 days of their other solid organ transplant using a renal allograft from the same donor. EPTS and KDPI were used to stratify kidney transplant recipients, and donors, respectively. Approval to conduct this analysis was obtained from the Thomas Jefferson University Institutional Review Board.

### Evaluating Organ-sharing Equity: Waitlist Time and Allograft Quality

We assessed organ equity in single and multi-organ transplants through multiple factors associated with organ availability and utilization. First, we analyzed waiting times for deceased donor kidney allograft, comparing median wait times to transplant of KTA recipients with those in each MOT category. We also examined how waiting times changed during the time period. Organ availability was then further examined by comparing the KDPIs of KTAs and MOTs. The distribution of KDPI was evaluated for KTAs and MOTs via histogram analyses and percentage of transplants using grafts from KDPI <35% donors. While KDPI has not been assessed or validated as a marker of donor quality in the non-renal setting, other non-renal organ-specific donor indices have been created that share similar components to KDPI.^12,13^ To normalize donors we assessed KDPI of isolated pancreas, liver, heart, and lung and transplants compared with their MOT counterpart, identifying the non-renal transplant KDPI values by identifying matched kidney donors within the OPTN database.

### Evaluating Transplant Frequency for Predialysis and Older Recipients

Access to kidneys for KTA and MOT was described through analysis of 2 distinct sub-populations of patients awaiting kidney transplant: (1) predialysis patients receiving preemptive transplants and (2) older patients aged >65 years old at transplant. Access in both populations was examined by comparing their proportions within each of the total MOTs and KTA populations. We also analyzed the proportions of these transplants using KDPI <35% kidneys.

### Comparing Kidney Graft Survival

We assessed kidney utilization in 2 contexts. To better categorize allograft survival in each transplant setting, we performed Cox proportional hazard regression modeling and Kaplan Meier curves, comparing survival in each category of MOTs to survival in KTAs stratified by EPTS. The relative value of using KDPI < 35% versus KDPI ≥ 35% in terms of graft survival was then assessed by comparing KTAs stratified by EPTS versus the MOT cohort. Finally, we examined the use of KDPI ≤ 20% and KDPI < 35% in KTA and MOT recipients looking at high-quality graft use frequency, waiting times, and kidney graft survival.

### Statistical Analysis

Continuous variables were evaluated for normality using the Shapiro-Wilk test. Non-normally distributed variables were compared with a Wilcoxon rank-sum test and represented as median (interquartile range). Categorical variables were compared using a chi-square (χ2) test and represented as number (percentage of population). Post-transplant patient and graft survival were reported graphically with Kaplan-Meier curves and numerically by time-varying Cox proportional hazards ratios (HRs) and 95% confidence intervals (95% CIs). Two-sided statistical significance was set a priori at *P* < 0.05. All statistical analyses were performed using Stata/MP 16.1 (Statacorp, College Station, TX).

## RESULTS

### Evaluating Organ-sharing Equity: Waitlist Time and Allograft Quality

We first compared median wait times to receive a kidney transplant (Table 1). From 2015 through 2019, the median waitlist time for 62,252 chronic renal failure patients receiving a KTA was 1,643 days. In comparison, 3,976 SPK recipients waited 586 days, SLK (n = 3,212), SHK (n = 808), and SOK (n = 73) recipients all waited a median time of <100 d. KTA recipients whose EPTS ≤ 20% had a 397-d shorter median waiting period than KTA recipients whose EPTS was >20%. To assess how waiting times evolved, transplants in each category were compared between calendar years 2015 and 2019. KTA recipients had a decrease in median waiting times of 260 days as well as SPKs whose median waiting times dropped 73 days and SHKs who decreased by 83 days. Conversely, SLK median wait times increased 41 days. SOK populations were too small for meaningful analyses.

**TABLE 1.** Median waitlist time in days for kidney transplant. A, All KTAs vs MOT types. B, KTA with EPTS > 20% vs KTA with EPTS≤20%

		Median time in days until kidney transplant (IQR)	*P* value vs all KTAs	2015	2019	*P* value 2015 vs 2019
				Waitlist time	Waitlist time	
A	All KTAs	1,643 (935–2,457)	-	1,824 (1,019–2,732)	1,564 (866–2,382)	<0.01
	SPK	586 (312–1,040)	<0.01	655 (354–1,203)	582 (313–1,020)	<0.01
	SLK	99 (23–370)	<0.01	72 (20–262)	113 (26–451)	<0.01
	SHK	82 (22–339)	<0.01	132 (29–405)	49 (9–377)	<0.01
	SOK	77 (15–281)	<0.01	360 (52–1,089)	91.5 (10.5–474)	0.13
B			*P* value KTA EPTS>20% vs KTA EPTS≤20%			
	KTAs >20%	1,740 (1,014–2,555)	-	1,927 (1,105–2,863)	1,652 (952–2,457)	<0.01
	KTAs ≤20%	1,343 (714–2,095)	<0.01	1,525 (828–2,291)	1,247.5 (625–2,007)	<0.01

**FIGURE 1.**
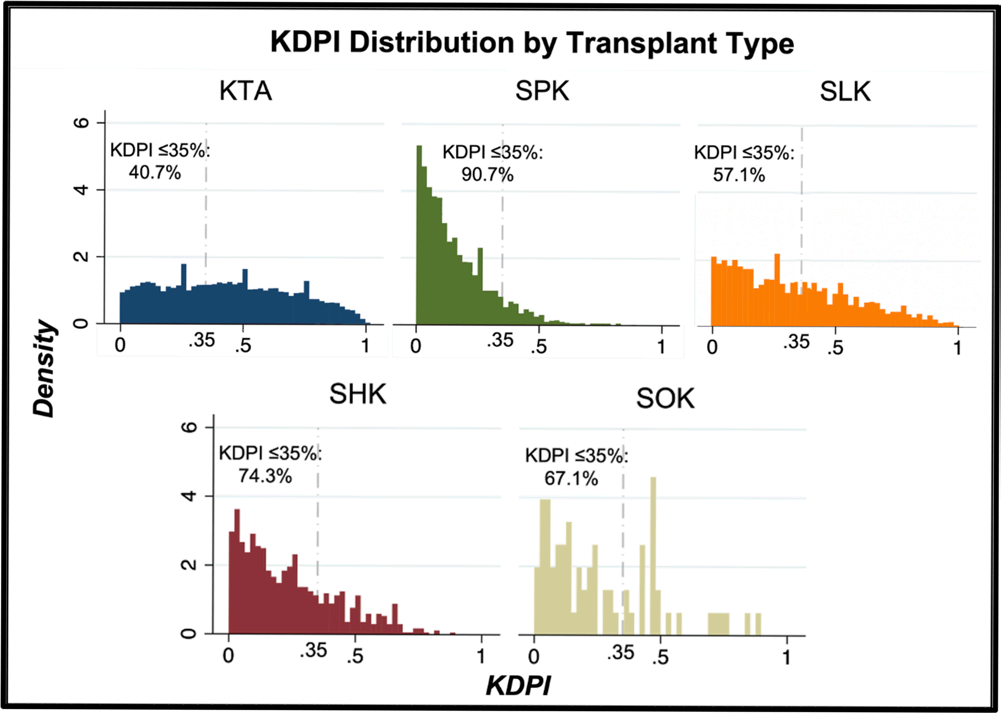
Histogram of KTA and MOT donor quality by KDPI distribution. KDPI, Kidney Donor Profile Index; KTA, kidney transplant alone; MOT, multiorgan transplants utilizing kidneys.

We then assessed the quality of renal allograft each subpopulation received when transplanted. Through a histogram analysis of donor KDPI (Figure 1), allograft quality was relatively widely distributed in the KTA population (median KDPI 43%, interquartile range: 22%–65%). Each MOT population, comparatively, demonstrated varying degrees of left skew, indicating lower KDPI allografts. Median KDPI was 12% (6%–23%) for SPKs (*P* < 0.01), 29% (13%–51%) for SLKs (*P* < 0.01), 20% (9%–36%) for SHKs (*P* < 0.01), and 20% (9%–43%) for SOKs. When comparing the use of high-quality allografts as defined by KDPI <35%, MOTs were again advantaged. KTAs utilized high-quality grafts in 39.56% of transplants (n = 24,625), with 43.61% (n = 10,738) of these grafts going into EPTS≤20% recipients, compared with 89.81% of SPKs, 55.67% of SLKs, 73.89% of SHKs, and 67.12% of SOKs (Table 2).

**TABLE 2.** Kidney availability and access for predialysis and elderly ESRD patients. A, Total number of different types of deceased donor kidney transplants and the number of KDPI < 35% kidney transplants. B, Frequency of predialysis transplants and use of KDPI < 35% donors in these predialysis recipients. C, Frequency of >65-y-old kidney transplants and use of KDPI<35% donors in these older recipients

(A) All transplants	KTA	SPK	*P*	SLK	*P*	SHK	*P*	SOK	*P*
Total number of transplants	62,252	3,976		3,212		808		73	
Number of KDPI <35% transplants	24,625 (39.56%)	3,571 (89.81%)	<0.01	1,788 (55.67%)	<0.01	597 (73.89%)	(<0.01)	49 (67.12%)	<0.01
(% of all transplants)									
(B) Predialysis kidney transplants									
All predialysis kidney transplants	4,545 (7.30%)	614 (15.44%)	<0.01	1,181 (36.77%)	<0.01	508 (62.87%)	<0.01	45 (61.64%)	<0.01
(% of all transplants)									
Predialysis recipients receiving KDPI <35% graft	2,013 (8.17%)	543 (15.21%)	<0.01	675 (37.75%)	<0.01	381 (63.18%)	<0.01	33 (67.35%)	<0.01
(% of KDPI <35% transplants)									
C) Kidney Transplants into 65yo.									
Number of Age >65 recipients	13,486 (21.66%)	21 (0.53%)	<0.01	732 (22.79%)	0.14	185 (22.90%)	0.39	2 (2.74%)	<0.01
(% of all transplants)									
Age >65 recipients receiving KDPI<35% transplant	2,916 (11.84%)	18 (0.50%)	<0.01	397 (22.20%)	<0.01	135 (22.61%)	<0.01	2 (4.08%)	0.12
(% of KDPI <35% transplants)									

We then compared single-organ non-renal transplants to their MOT counterparts (Table 3). Most notably, liver, heart and lung-alone transplants were much more common than their MOT counterpart. For liver transplantation, SLKs made up 12.5%; for heart transplantation, SHKs made up 7.1%; and for lung transplantation, simultaneous lung kidneys made up only 0.3%. The inverse was true with pancreas transplantation where SPKs made up 88.4% of the transplants. Pancreas-alone and heart-alone transplants had a slightly higher median KDPI values when compared with SPKs and SHKs. Much lower KDPIs were seen in SLKs (29%) compared with liver-alone transplants (37%). The small number of simultaneous lung kidneys prevented a statistically meaningful comparison for lung transplants.

**TABLE 3.** KDPI comparison for isolated pancreas, liver, heart, and lung transplants vs MOT counterpart

	Single organ		Multi organ (including kidney)		
	Number	KDPI % (IQR)	Number	KDPI % (IQR)	*P*
Pancreas	525	13 (6–26)	3,996	12 (6–23)	0.01
Liver	23,621	37 (16–62)	3,361	29 (13–51)	<0.01
Heart	11,785	21 (9–39)	834	20 (9–37)	<0.01
Lung	10,312	26 (11–50)	33	18 (7–43)	0.15

### Evaluating Kidney Allograft Access in Special Populations: Predialysis and Older Recipients

We analyzed 2 specific groups of patients on different ends of the allocation spectrum to assess equity in organ sharing. The first involved those who had not yet progressed to dialysis receiving preemptive kidney transplants. As shown in Table 2, 7.30% of all KTA recipients were predialysis. In comparison, predialysis recipients comprised 15.44% of SPKs, 36.77% of SLKs, and >60% of SHKs and SOKs. The second population assessed were older (age >65) recipients, which represented 21.66% of all KTA recipients (Table 2). This was similar to the proportion of SLK and SHK recipients. SPKs and SOKs had few elderly recipients.

We then looked at the use of KDPI <35% kidneys (Table 2). The proportion of predialysis recipients getting a KDPI <35% kidney was similar to its related cohort’s transplant rate (Table 2). This changed with >65-y-old recipients who represented 21.66% of the entire KTA population but only 11.85% of KTAs using KDPI <35% grafts. Thus, for KTA recipients >65 <22% received a KDPI<35% kidney. For the age >65 recipients all MOTs maintained a high frequency use of KDPI<35% kidneys. This is best illustrated by the SHK >65 recipients were 73% (135/185) received a kidney from a donor with a KDPI<35% (Table 2).

### Comparing Kidney Utilization Through Kidney Graft Survival

**FIGURE 2.**
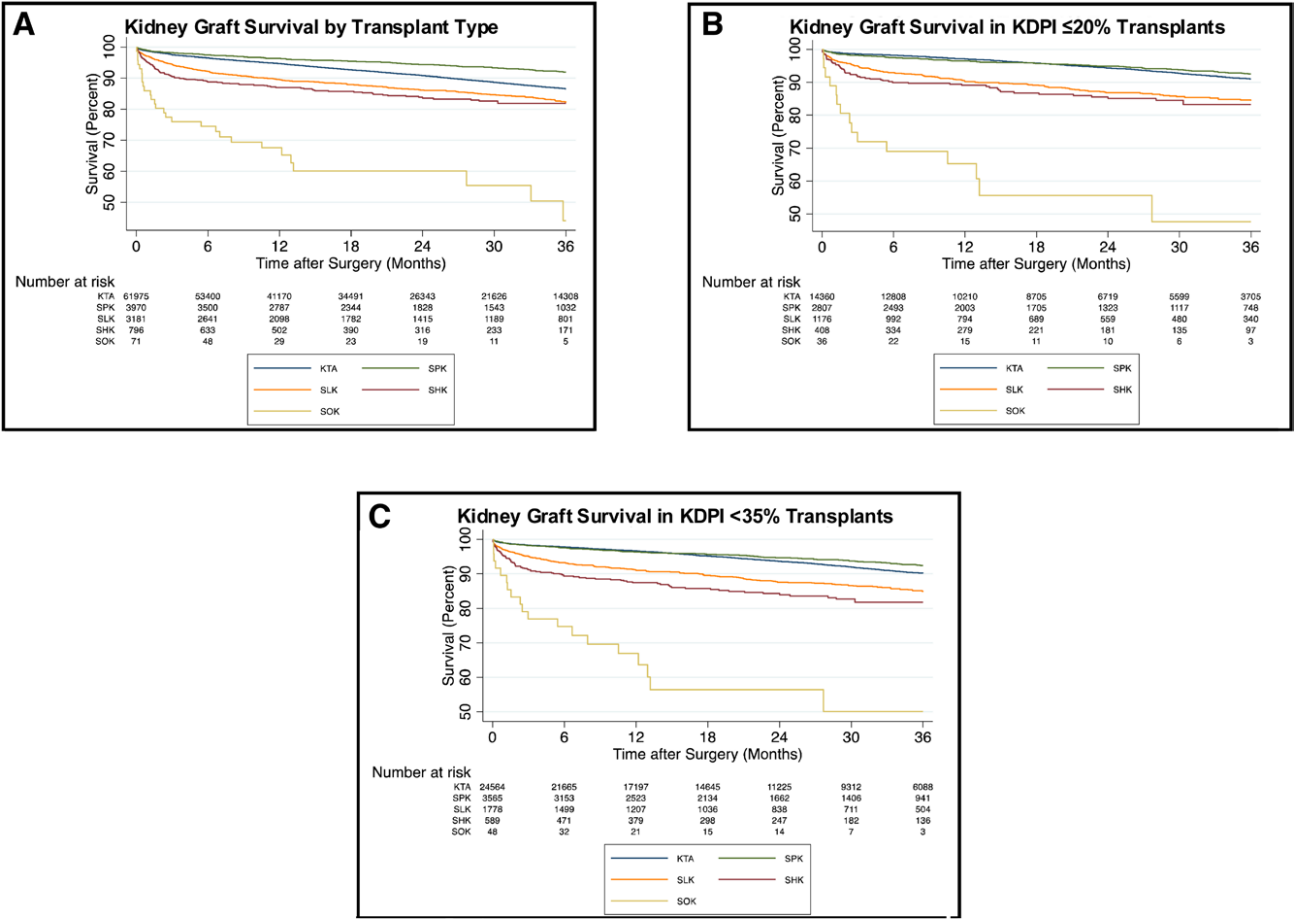
Kidney Graft Survival in various Transplant Scenarios. A, Kidney graft survival by transplant type. B, Kidney graft survival using allografts from KDPI ≤20% donors. C, Kidney graft survival using allografts from KDPI <35% donors. KDPI, Kidney Donor Profile Index.

Outcomes for MOT categories were then compared with KTA recipients. As demonstrated in Figure 2A, overall kidney graft survival for KTA recipients was inferior to kidney graft survival in SPK recipients except for EPTS≤20% recipients who had near equivalent survival (Table 4). Kidney graft survival was superior in KTAs compared with SOKs at all EPTS levels. KTAs with an EPTS ≤80% had better kidney graft survival than all SLKs and SHKs. SLKs did have better kidney graft survival than KTAs with an EPTS>80%. SHKs and EPTS>80% KTAs had near equivalent kidney graft survival.

**TABLE 4.** Kidney graft survival in EPTS-stratified KTA vs EPTS-independent MOT recipients

	Graft survival HR (95% CI)				
	KTA Number (% total KTA)	SPK	SLK	SHK	SOK
KTA EPTS ≤20%	14,517 (23.31%)	1.08 (0.95–1.24)	0.46 (0.41–0.51)	0.39 (0.33–0.47)	0.10 (0.07–0.15)
KTA EPTS 21-80%	33,449 (53.72%)	1.51 (1.33–1.71)	0.64 (0.58–0.71)	0.55 (0.46–0.66)	0.15 (0.11–0.22)
KTA EPTS >80%	14,286 (22.95%)	2.65 (2.34–3.02)	1.12 (1.01–1.24)	0.97 (0.81–1.16)	0.27 (0.18–0.39)

To assess the relative value of higher quality kidney allografts, graft survival for transplants using KDPI <35% and ≥35% donors were compared within each category. KDPI<35% was associated with improved kidney graft survival in the overall KTA cohort and this was also the case when KTAs were divided into 5 EPTS strata (Table 5). SPK and SLK both benefited from using KDPI<35% kidneys, while no significant difference kidney graft survival was observed for KDPI <35% donors in SHKs or SOKs.

**TABLE 5.** Comparison of kidney graft survival outcomes KDPI < 35% vs KDPI ≥ 35%

KDPI <35% vs ≥35%	HR	95% CI
KTA	0.57	0.55–0.61
EPTS ≤20%	0.67	0.59–0.76
EPTS 21-40%	0.69	0.60–0.80
EPTS 41-60%	0.65	0.56–0.76
EPTS 61-80%	0.62	0.54–0.71
EPTS >80%	0.71	0.64–0.79
SPK	0.61	0.43–0.86
SLK	0.71	0.59–0.86
SHK	0.84	0.55–1.30
SOK	0.98	0.45–2.19

We additionally compared access to high-quality kidneys and their post-transplant outcomes in KTA and MOT recipients. Using a KDPI cutoff of ≤20% (Table 6A) we noted that 23.12% of KTA recipients were transplanted with kidneys from KDPI ≤20% donors, with >62% of these (n = 9,022), going into EPTS≤20% recipients, as part of longevity matching. This was less frequent than SPKs (70.75%), SLKs (36.77%), SHKs (51.24%), and SOKs (50.68%). KTA candidates receiving KDPI ≤20% grafts waited a median of 1438 d on the waitlist and this shorter waiting time was driven by improved access for EPTS≤20% who had a median wait time of 1,345 days. In comparison, SPKs waited a median of 602 days, SLKs 71 days, SHKs 93 days, and SOKs 58 days. When using these grafts, kidney allograft survival in KTAs was comparable to SPKs and significantly greater than SLKs, SHKs, and SOKs (Figure 2B).

**TABLE 6.** Median waitlist time and kidney graft survival in KTA vs. MOT recipients for (A) KDPI ≤20% and (B) KDPI <35% allografts

*(A) KDPI ≤20% kidney transplants*													
	KTA	SPK	*P*	HR	SLK	*P*	HR	SHK	*P*	HR	SOK	*P*	HR
				(95% CI)			(95% CI)			(95% CI)			(95% CI)
Number of transplants using KDPI ≤20% grafts	14,391	2813	<0.01	-	1181	<0.01	-	414	<0.01	-	37	<0.01	-
% of all transplants	23.12%	70.75%			36.77%			51.24%			50.68%		
Waitlist days (IQR)	1438	602	<0.01	–	71	<0.01	–	93	<0.01	–	58	<0.01	–
	(805–2201)	(313–1069)			(17–287)			(25–340)			(23–281)		
Kidney graft survival	–	–		0.88	–	–	1.94	–	–	2.21	–	–	9.68
				(0.75–1.03)			(1.64–2.29)			(1.69–2.87)			(5.81–16.13)
*(B) KDPI <35% kidney transplants*
	KTA	SPK	*P*	*HR*	SLK	*P*	*HR*	SHK	*P*	*HR*	SOK	*P*	*HR*
				*(95% CI*)			*(95% CI*)			*(95% CI*)			*(95% CI*)
Number of transplants using KDPI <35% grafts	24,625	3571	<0.01	–	1788	<0.01	–	597	<0.01	–	49	<0.01	–
% of all transplants	39.56%	89.81%			55.67%			73.89%			67.12%		
Waitlist days (IQR)	1580	586	<0.01	–	99	<0.01	–	82	<0.01	–	77	<0.01	–
	(886–2392)	(312–1040)			(23–370)			(23–339)			(24–195)		
Kidney graft survival	–	–		0.81	–	–	1.69	–	–	2.28	–	–	8.33
				(0.70–0.93)			(1.47–1.94)			(1.85–2.81)			(5.30–13.10)

Using a KDPI cutoff of <35% (Table 6), KTA recipients of KDPI <35% grafts waited a median of 1,580 days on the waitlist, compared with 586 days for SPK, 99 days for SLK, 82 days for SHK and 77 days for SOK. When using these grafts, kidney allograft survival in KTA was slightly inferior to SPKs and superior to SLKs, SHKs, and SOKs (Figure 2C).

## DISCUSSION

The main purpose of this study was to characterize the relative access and outcomes of deceased donor kidney allografts in MOT compared with chronic renal failure KTA recipients. This was done through a retrospective review of the OPTN database examining trends in transplantation for KTA and MOT patients since the implementation of KAS. We found that KTA recipients waited significantly longer and received a kidney with a higher KDPI than any MOT category. Upon assessing predialysis and elderly recipients, we found that predialysis patients had better access to kidney transplantation when paired with a non-renal organ. In addition, the quality of these kidneys again was superior in the MOTs. Among recipients >65 year old, there were only 20 SPKs and SOKs, but the proportion of SLKs and SHKs made up more than 20% of recipients of these types of transplants. Even among the elderly recipients, MOTs access to kidneys with a KDPI < 35% was preserved, whereas older KTA recipients received a KDPI<35% much less frequently. Comparing kidney graft survival in MOTs to KTA recipients, we observed different outcomes based on MOT type. SPKs and KTAs with an EPTS ≤ 20% or KTAs with a KDPI≤20% tended to have the best graft survival. KTAs with an EPTS ≤ 80% fared better than SLKs, SHKs, and SOKs. EPTS ≤ 20% KTA recipients did wait nearly 400 days less than EPTS >20% KTAs, but their waiting time remained substantially longer than all MOTs. Graft longevity was dependent on KDPI for KTA, SPK, and SLK transplants while using KDPI<35% kidneys did not prolong kidney graft survival in SHK and SOK settings. The smaller sample sizes in the SHK and SOK groups, combined with a KDPI C-statistic of only 0.68,^14^ suggest that this is a consequence of underpowered data. In addition, KDPI reflect outcome estimates based on KTA recipients alone, and this tool likely loses accuracy when combined with other organ transplants. Our findings suggest that directing more low KDPI kidneys towards KTA candidates with an EPTS≤80% could further enhance utility and equity in kidney transplantation.

One of the principal drivers of deceased donor kidney access for chronic renal disease candidates is their duration on the waitlist. Before KAS, waitlist duration could only start after listing by a transplant center. This resulted in marked disparities in access to kidney transplantation, as patients with marginal access, if they were lucky enough to be listed for a deceased donor kidney, were ultimately listed later in their disease course, and thus also waited longer to receive a kidney transplant.^15,16^ Pre-KAS, there were no policies on the adult candidate side that considered donor/recipient longevity matching. Roughly 50% of the deceased donor kidney transplants done at that time had a donor-to-recipient age discrepancy of >15 years.^17^ Thus, one of the issues with the pre-KAS system was a large percentage of kidney transplant recipients dying with a renal allograft that still had many more years of potential function.^18^ KAS directly improved equity by allowing the candidate who was referred after initiation of dialysis, to capture all dialysis time, as waiting time. From a kidney quality standpoint, KAS relied on the more granular KDPI score to define 4 different allocation algorithms used to place a kidney. This was clearly superior to the pre-KAS standard criteria donor versus expanded criteria donor buckets. KAS also improved access for certain sensitized groups (19%<CPRA<80% and CPRA>84%) and blood group B candidates, while leveling some of the access for candidates who were unfairly advantaged in the pre-KAS system (CPRA 80%–84%).^17^ Subsequent research has shown that KAS has been able to significantly reduce the time to receive a kidney transplant for many socioeconomically disadvantaged populations.^19^ Despite transplanting a larger fraction of highly sensitized and socioeconomically disadvantaged candidates, KAS did preserve post-transplant graft survival.^20^

KAS failed to address certain problems and made some issues worse. Geographic disparity considerations based largely on Organ Procurement Organizations (OPO) boundaries were not addressed by KAS.^21^ In December 2019, the OPTN board eliminated donor-specific service areas (OPO boundaries) from deceased donor kidney allocation in favor of 250 nautical mile circles drawn around the donor hospital. The transplants examined in our study precedes the implementation of this geographic policy. In the area of nonutilization of deceased donor kidneys, KAS sought to improve utilization of high KDPI (>85%) grafts by introducing regional allocation of these grafts. While kidney graft travel increased with the implementation of KAS, so did the nonuse rate, particularly from high KDPI (>85%) donors.^22,23^ Thus, deceased donor kidney nonuse is one of the biggest challenges facing the United States transplant system today.^22,24^

While KAS addressed several equity and utility issues, allocation of renal allografts to MOT recipients exists outside of the KAS. In each MOT class, policy is dictated by the non-renal organ. Pancreas transplantation remains distinct from other MOT categories. The improvements in insulin delivery and glucose sensing technologies have improved therapy for diabetics and impact the risk-benefit decision associated with pancreas transplantation.^25^ The pancreas graft is highly sensitive to physiologic insults that may precipitate ischemia-reperfusion injury,^26^ and thus deceased donors are carefully selected. Thus, SPK transplant rates have not increased in the United States with 909 SPK transplants occurring in 2000, and 827 occurring in 2020. This lack of growth is markedly different than all other US organ transplant types where growth in transplant numbers is the norm.^27,28^

Studies assessing the utility of SLK over liver transplant alone have demonstrated a benefit in select patients with renal failure.^29,30^ Between 2008 and 2017, SLK rates increased annually, comprising 5.3% of all deceased donor renal allograft usage during the 2017 calendar year. Responding to this rise, in August 2017, the OPTN adopted a policy defining specific criteria for SLK use. In addition, a “Safety Net” was created for post-liver transplant recipients with ongoing renal failure.^31^ This policy provided guard rails for defining who are actual candidates for SLK transplants. It also included priority listing after liver transplant if the candidate remained in post-transplant renal failure and was otherwise a suitable candidate for kidney transplantation.^32-34^ The numbers of liver-kidney transplants nationally did decrease with enactment of this policy, with SLK representing 8.75% of deceased donor liver transplants before policy implementation in 2017 and dropping to 7.92% in 2018. In subsequent years, SLK rates have hovered just above 8% of all deceased donor liver transplants. Of the 112 kidney transplant recipients meeting Safety Net criteria in our data set, 78 received KDPI < 35% kidneys and only 2 received kidneys with a KDPI >85%. Graft survival has been excellent, with 98.21% functioning at 3 y. While the SLK allocation policy appears to have interrupted the continued rise in SLK transplants while successfully utilizing the Safety Net to direct kidneys to patients with renal failure following liver transplant,^35^ both require ongoing surveillance to help guide future policy.

Similar to SLKs, SHK transplantation has increased in frequency, substantiated by several studies showing improved overall survival for heart transplant recipients with diminished glomerular filtration rates.^36^^,^^37^ At least 1 study did not see an overall survival benefit in kidney transplantation for SHK recipients who were not dialysis dependent.^38^ But a more recent study using OPTN data suggests that SHK recipients may have a survival benefit with pre-transplant eGFR of up to 40 mL/min/1.73m^2^, even though 1-y kidney graft failure was 3 times higher in this group compared with the mate kidney used in a chronic kidney failure recipient.^8^ In our study, the non-dialysis SHK population represented 63% of all SHKs and used a KDPI<35% kidney 63% of the time as well. SHK rates have increased by 483% from 2010 to 2021 and currently utilize nearly 2% of the available deceased donor kidney pool. In June of 2022, the OPTN board approved a policy change in heart kidney allocation analogous to the policies for SLK, with implementation as of September 28, 2023. Thus far, adoption of an SHK (as well as a simultaneous lung-kidney (SLuK)) allocation policy in addition to a Safety Net for both has resulted in less multiorgan transplants, but more patients being registered as eligible for SHK (or SLuK) since adoption of the new policy.^11^ This has been theorized to be a potential consequence of incorporating the more chronically ill Status 4 and 5 heart transplant waitlist candidates that may have concurrent chronic kidney disease. Overall, the policy seems to be achieving the same affect seen with SLK, although the increase in registrants warrants further observation particularly with regard to rates of Safety Net kidneys in post-heart transplant recipients moving forward.

While there are immunologic benefits to utilizing one common donor compared with 2 in MOT,^39^ our analyzed data was likely affected by transplant teams feeling pressure to use the same donor kidney in suboptimal physiologic circumstances and this probably has played a role in worsening SLK, SHK and SOK kidney graft survival. It seems likely that safety net policies after liver and heart transplant will improve kidney graft outcomes in these settings. Transplant teams no longer feel pressure to use the same donor kidney if the liver or heart recipient is not in suitable physiologic shape for the kidney graft. However, the safety net itself is a further drain of kidneys away from the standard chronic renal failure KTA candidates. One protection for the EPTS≤20% KTA candidate group, is that the advantaged position on the deceased donor kidney waitlist for the liver and heart recipients awaiting a safety net kidney, does not include KDPI ≤ 20% kidneys. Nevertheless, in the data we examined the majority of Safety Net liver recipients received a kidney graft with a KDPI < 35%.

Our study has several limitations. We used a large nationally maintained data source that has high numbers but lacks granularity.^40^ We did not examine mortality or delisting for KTA or MOT candidates. It is recognized that for many areas of the United States, the median time to transplant for deceased donor kidney candidates is not reportable because the median candidate is more likely going to be delisted rather than receive a deceased donor kidney transplant.^41^ Our study also did not examine data from a deceased donor kidney nonuse perspective which is a major problem. Policy development in solid organ transplantation is being directed towards a continuous distribution for all organ allocations, and this has already been operational for lung allocation.

Deceased donor renal allograft allocation is a topic of tremendous complexity with no clearly defined answer. Transplant practices in the United States have strongly encouraged use of low KDPI kidneys into MOTs, even though, that with exception of SPKs, these kidney grafts did not do as well as they would if that kidney was directed to a chronic renal failure KTA candidate. It seems unlikely that the specific criteria and safety net policies for SLK and SHK will dramatically change this. It does appear that longevity matching through KAS’s KDPI≤20% into EPTS≤20% has been successful in getting these candidates transplanted sooner with better quality organs. The introduction of continuous distribution, which can factor in longevity matching along with medical urgency, anticipated waitlist survival, geography, and hierarchical modeling of MOT allocation may help economize and improve kidney allograft utilization, and the OPTN MOT Committee has done substantial work to help understand where MOT allocation will fit into a continuous distribution model. To address the current national kidney allograft shortage, however, this work will need to function in tandem with significant efforts to optimize candidate selection, increase deceased donor identification, utilization, and yield per donor, improve organ preservation, and increase living donor transplantation. OPTN goals remain working towards an encompassing continuous distribution in which each individual kidney is allocated based on equity, disease burden, and societal benefit. This study, along with the existing and future bodies of literature on the matter, should provide valuable information to allow for maximal utilization of an invaluable and scarce resource.
